# Supplementary material for: Uncovering the Secrets of Prostate Cancer’s Radiotherapy Resistance: Advances in Mechanism Research
Source: Biomedicines. 2023 Jun 3;11(6):1628. doi: 10.3390/biomedicines11061628 (PMC10296152; doi:10.3390/biomedicines11061628)
Supplement: Supplementary file 1 [file biomedicines-11-01628-s001.zip › biomedicines-2376072-supplementary.pdf]

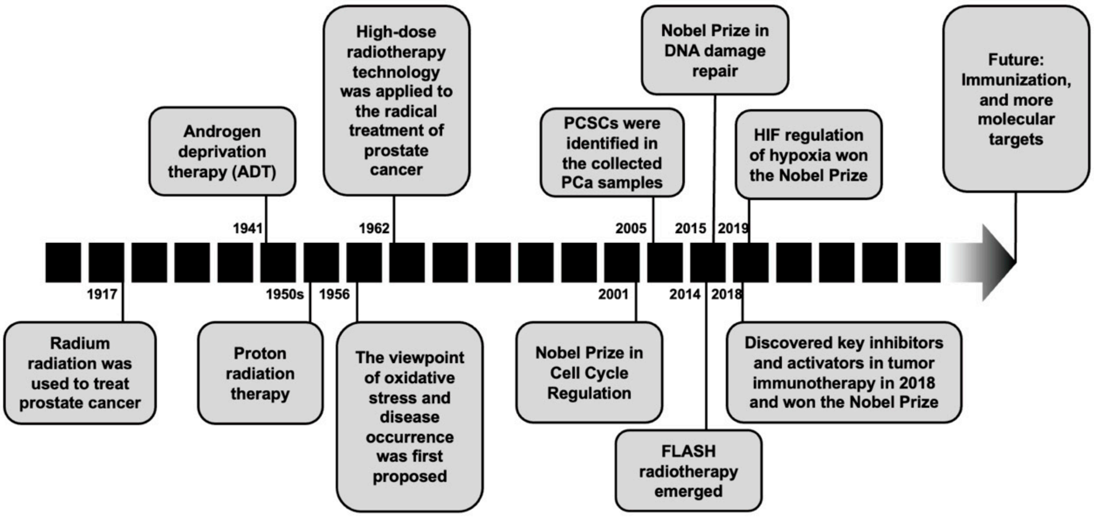

Figure S1. Timeline of important radiotherapy techniques and biological processes studied in radiotherapy resistance of prostate cancer.

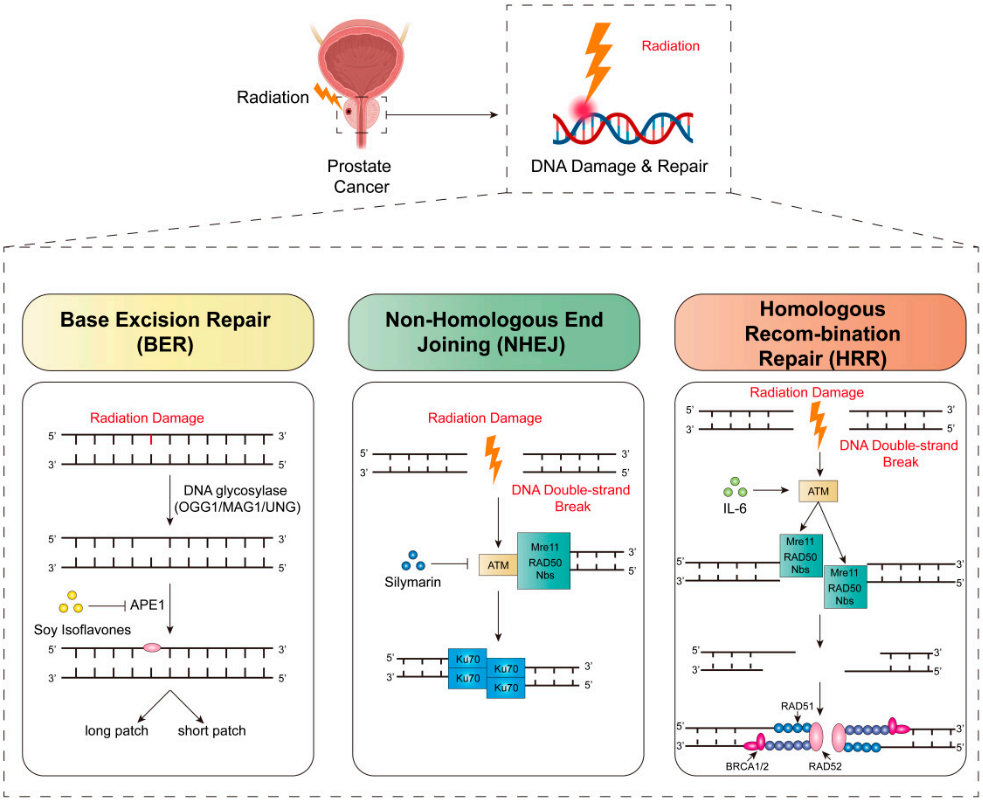

Figure S2. Targeted therapeutic agents and bio-activated compounds: unveiling their specific role links in the DNA damage and repair pathways.

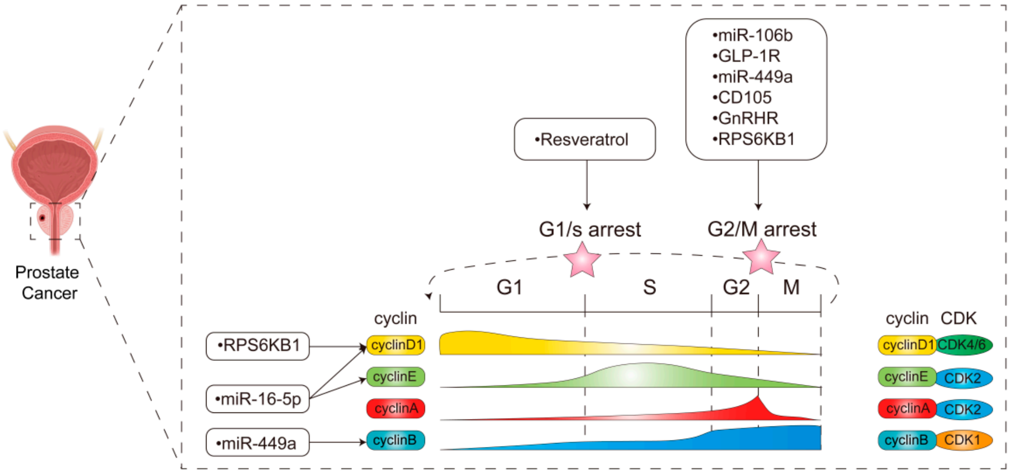

Figure S3. Targeted therapeutic agents and bio-activated compounds: unveiling their specific role links in the cell cycle.
